# Secondhand Smoke Exposure and Cognitive Function in U.S. Never-Smoking Older Adults

**DOI:** 10.1093/geroni/igaf122.2092

**Published:** 2025-12-31

**Authors:** Sophie Zhijing Xu, Jinyu Hu, Jackie Finik, Ruotong Liu, Yaguang Zheng, Bei Wu

**Affiliations:** New York University, New York, New York, United States; New York University, New York, New York, United States; New York University, New York, New York, United States; New York University, New York, New York, United States; New York University, New York, New York, United States; NYU Shanghai, Shanghai, China (People’s Republic)

## Abstract

While active smoking is a known risk factor for cognitive decline, the impact of secondhand smoke (SHS) on cognitive function in never-smoking adults remains understudied. This study examines the association between SHS exposure and cognitive function in U.S. never-smoking older adults using data from 615 individuals aged 60+ from the NHANES 2011–2014. SHS exposure was measured via serum cotinine levels and categorized as low (0.01–0.02 ng/mL), moderate (0.02–0.04 ng/mL), and high (≥0.04 ng/mL), with never-smokers defined as those reporting fewer than 100 lifetime cigarettes. Cognitive function was assessed using the CERAD-WL immediate and delayed recall, Animal Fluency Test, Digit Symbol Substitution Test (DSST), and Global Cognitive Score. Multivariable regression models adjusted for demographic, lifestyle, and socioeconomic factors. The mean age was highest in the unexposed group (69.50, SD = 6.69 years), followed by the low, moderate, and high exposure groups. Moderate SHS exposure was significantly associated with lower scores in CERAD-WL immediate recall (β = -1.94, p < 0.05), delayed recall (β = -0.87, p < 0.05), DSST (β = -3.81, p < 0.05), and Global Cognitive Score (β = -0.31, p < 0.01), while no significant associations were observed in the low and high exposure groups. These findings suggest that even moderate SHS exposure may contribute to worse cognitive performance in never-smoking older adults, emphasizing the need for public health efforts to minimize SHS exposure and preserve cognitive health in aging populations.

